# Evaluation of the Shear Performance of Douglas-Fir Wood at Elevated Temperatures

**DOI:** 10.3390/ma15238386

**Published:** 2022-11-25

**Authors:** Lingfeng Zhang, Qianyi Li, Weiqing Liu, Qian He, Yan Liu, Kai Guo

**Affiliations:** 1 College of Civil Science and Engineering, Yangzhou University, Yangzhou 225127, China; 2 Advanced Engineering Composites Research Center, Nanjing Tech University, Nanjing 211816, China; 3 Department of Architecture, Built Environment and Construction Engineering, Politecnico di Milano, Piazza Leonardo da Vinci 32, 20133 Milano, Italy

**Keywords:** Douglas-fir, wood, elevated temperature, shear, digital image correlation (DIC)

## Abstract

Shear fracture frequently occurs in timber beams and panels subjected to transverse loads. At elevated temperatures, wood will undergo complex physical and chemical processes which significantly affect the shear properties. In this paper, the v-notched Douglas-fir specimens with three different shear planes: (a) Radial-Tangential (RT); (b) Radial-Longitudinal (RL), and (c) Longitudinal-Radial (LR), were fabricated and tested under the elevated temperatures from 20 °C to 180 °C. The digital image correlation (DIC) technique was used to measure the shear strain. It was found that the shear plane had a significant effect on the failure modes, shear strength, and shear modulus. The shear strength and shear modulus generally decreased with the increase of temperature. However, the shear strength was significantly improved when the hardening of the dry lignin occurred between 100 °C and 140 °C. Moreover, the design curve for the shear strength in Eurocode 5 is conservative for all the specimens with different shear planes.

## 1. Introduction

Wood is increasingly used as the building material for construction due to the advantages, such as light weight, high parallel-to-grain strength, good acoustic property, good insulation, and natural aesthetic appreciation. Compared with its counterparts like steel and concrete, wood is renewable and biodegradable which attracts great interests of the civil engineers and researchers [1,2,3]. However, when subjected to elevated temperatures, wood will undergo the glass transition between 50 °C and 70 °C, followed by the dehydration (moisture evaporation) around 100 °C. After the evaporation of the water, the pyrolysis begins at 250 °C, and finally the wood is decomposed into char [4,5]. As a result, the mechanical properties of wood are significantly sensitive to elevated temperatures. One of the most important mechanical characteristics is the shear performance of wood. For example, shear connectors are frequently used in timber-concrete composite (TCC) beam for shear stress transfer. Moreover, the shear property of the wood has a significant effect on the mechanical behavior of the connections between the cross-laminated timber (CLT) members. Another example is that the composite deck consists of two glass-fiber reinforced polymer (GFRP) face sheets and the Balsa core, commonly exhibiting a typical shear failure of wood core when subjected to transverse bending load [6,7].

The shear properties of wood have been investigated by several experimental and analytical studies. Liu and Floeter [8] developed a formula for shear strength of orthogonal materials and the formula was verified through the shear tests of spruce. Gupta and Sinha [9] studied the effect of grain angle on the shear strength of Douglas-fir wood. As the angle increased from 0° (Longitudinal-Tangential) to 90° (Longitudinal-Radial), the average shear strength decreased from 6.57 MPa to 2.24 MPa. Further, a new formula is proposed to predict the shear strength of the Douglas-fir wood with different angles. More recently, the Arcan shear tests on the Pine wood were conducted by Bilko et al. [10]. The digital image correlation (DIC) technique was used to measure the shear modulus of the Pone wood. Moreover, the developed finite element model can arcuately predict the experimental failure modes.

Elevated temperatures have a significant effect on the mechanical properties of wood. Hence, the safe design and construction of timber structure require an understanding of the mechanical performance of wood under elevated temperatures. Goodrich et al. [11] studied the compressive strength and thermal softening of end-grain balsa wood at elevated temperature (20–300 °C). It was found that the compressive strength in axial and radial directions decreased linearly with increasing temperature. At 250 °C, the compressive strength decreased to 20% of the room temperature. Moreover, the degradation of the compressive strength at 250 °C can be recovered after cooling down. This was because the degradation was mainly dominated by the reversible glass transition of the hemicellulose and lignin. The parallel-to-grain compressive strength of the Paricá wood subjected to elevated temperatures from 20 °C to 230 °C was measured by Manríquez and Moraes [12]. It was found that the compressive strength decreased nonmonotonically, exhibiting a 35% reduction in compressive strength at 230 °C. The bending performance of Chinese Larch wood was reported by Zhong et al. [13]. It was found that the bending strength and the modulus of elasticity reduced nonlinearly with the increase in temperature.

Law and Koran [14] conducted the torsional-shear experiments of five softwoods and four hardwoods subjected to elevated temperatures from 22 °C to 150 °C. It was found that the reduction in shear stress is independent on the physical and structural wood species at the elevated temperature. Cylindrical specimens were adopted by Dhima et al. [15] to conduct shear tests of glulam specimens at elevated temperatures. This experiment showed that the wood moisture content had no significant effect on the shear strength, but on the failure mode. The experimental strength degradation was compared with the design curve in EN 1995-1-2 (Eurocode 5, EC 5) [16]. It was found that the design curve given by EC 5 remains conservative. Recently, the influence of elevated temperature on the shear performance of end-grain balsa wood, was experimentally and analytically investigated by Garrido et al. [17]. The results indicated that all specimens exhibited non-linear shear stress–strain behavior and the non-linearity characteristic became more obvious with increase of temperature. The shear strength and shear stiffness of the Balsa wood both reduced more than 80% (of that at the room temperature) at 240 °C. Yue et al. [18] studied the tensile, compressive, and bending properties of Douglas-fir under nitrogen atmosphere at different temperatures. The retention rates of the compressive, tensile, and bending strengths at 280 °C under nitrogen were 22%, 5%, and 23%, respectively. From SEM observation, the authors found that the microstructural damage of the cell wall occurred at 280 °C.

It can be seen that the existing investigations on the shear properties of wood are mainly conducted at the ambient temperature. The investigation of shear performance of wood at elevated temperatures is still limited. Moreover, the influence of shear plane on the shear behavior of wood has not been well known. This study aims to investigate the shear performance of the Douglas-fir wood at elevated temperatures. Three types of shear specimens with different shear planes were designed and tested by using of Iocipescu method from 20 °C to 180 °C. The DIC technique was used to capture the temperature-dependent shear strains [19]. Finally, the temperature-dependent shear strength and shear modulus were presented and discussed.

## 2. Experimental Program

### 2.1. Materials

The North America Douglas-fir sapwood with an age around 35 years (supplied by the Interfor Corporation, Vancouver, BC, Canada) was used in the experiments. The density ranges from 470 to 530 kg/m^3^ according to ASTM D2395 [20]. The average moisture content was 11.8% according to ASTM D4442 [21]. The parallel-to-grain tension and compression tests were conducted according to ASTM D143 [22]. The average parallel-to-grain tensile and compressive strength were 59 MPa and 48 MPa, respectively. The average parallel-to-grain tensile modulus was 11.5 GPa.

### 2.2. Specimens

**Figure 1:**
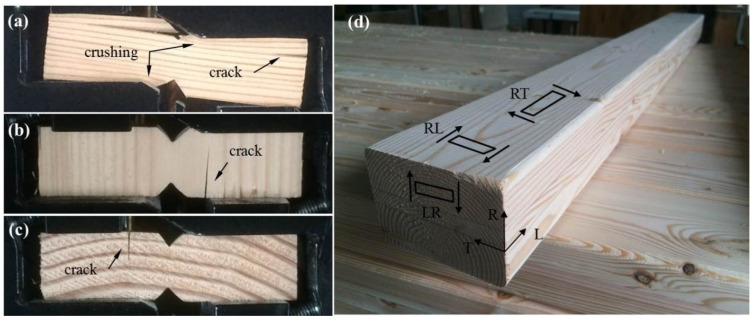
Premature failure in specimens without tabs at room temperature: (**a**) RT; (**b**) RL; (**c**) LR; (**d**) Illustration of shear plane.

**Figure 2:**
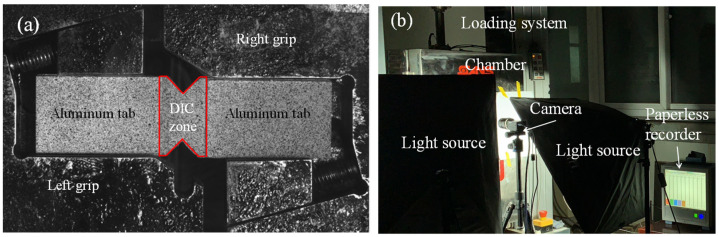
Test setup: (**a**) specimen configuration with DIC zone and (**b**) test systems.

The specimens with the v-notches were cut from lumbers according to ASTM D5379 [23]. Figure 1 shows the configurations of the iosipescu specimens with three shear planes: Radial-Tangential (RT), Radial-Longitudinal (RL), and Longitudinal-Radial (LR). The length, width and the thickness for all specimens are 76 mm, 19 mm and 5 mm, respectively. The tests are divided into three series according to the shear planes. For each series, a total of 40 specimens (5 specimens per temperature) were examined at eight target temperatures (20, 40, 60, 80, 100, 120, 140, and 180 °C). The specimens with RT, RL, and LR shear planes were respectively labeled as RT-x, RL-x and LR-x, with x being the temperature. The preliminary tests showed that the all the specimens were failed with premature failure modes, as shown in Figure 1. Hence, to avoid stress concentration and local crushing, four aluminum tabs with a thickness of 1.5-mm were bonded to the end of the specimen, as shown in Figure 2a. The tested specimens are summarized in Table 1.

### 2.3. Iosipescu Shear Test Set-Up

As shown in Figure 2, the specimens were placed on the test fixture before heating in the environmental chamber. During the shear experiment, the two-dimensional (2D) DIC system was used to measure the deformations and strains of the specimen under elevated temperatures. In order to measure the strain filed, the speckle patterns on the specimens were artificially made by spraying black paints on the surface preprinted with white lusterless paint, as shown in Figure 2a. The commercial PMLAB DIC system, consisting of one high-resolution camera (GRAS-50S5C, supplied by Point Grey Research Inc, Vancouver, BC, Canada) with a resolution of 2448 pixels × 2048 pixels, a tripod, and the FLYCAPTURE SDK camera control software, was used to capture the images at a rate of 1 Hz during the test. The camera is placed in front of the environmental chamber with its optical axis perpendicular to the specimen surface. The PMLAB DIC-3D software was used to calculate the 2D deformations and strains of the notched central part of the specimens.

The test was conducted by three steps. Firstly, the test specimen and a reference specimen with thermocouple (K type) embedded inside the center (for measuring the specimen temperature) were placed in the chamber. Two metal spindles with round mental bearing plates inside the chamber served as the actuator and supporter. In the second step, the chamber was heated form the room temperature (15 °C to 20 °C) to the predefined temperature and the center temperature of the specimen was measured by the paperless recorder (GP20, supplied by Yokogawa China Co., Ltd., Shanghai, China). After the center of the specimen reached the predefined temperature, the specimen in the chamber was kept at the predefined temperature for 5 min before testing. Finally, the specimen was loaded by the universal machine at a speed of 1 mm/min up to failure while the images of the specimen were captured by the DIC system. Figure 2b shows the illustration of setup for the Iosipescu shear test.

### 2.4. Thermogravimetric Analysis

In order to study the thermal decomposition process of the Douglas-fir wood, thermogravimetric analysis (TGA) tests were carried out under air atmosphere from room temperature to 600 °C. The Douglas-fir powder was used as the test sample, and the heating rates were 5, 10, and 20 °C/min. A NETZSCH STA409 analyzer was used to measure the relationship between the mass fraction and temperature during the heating process.

## 3. Experimental Results and Discussions

### 3.1. TGA Results

**Figure 3:**
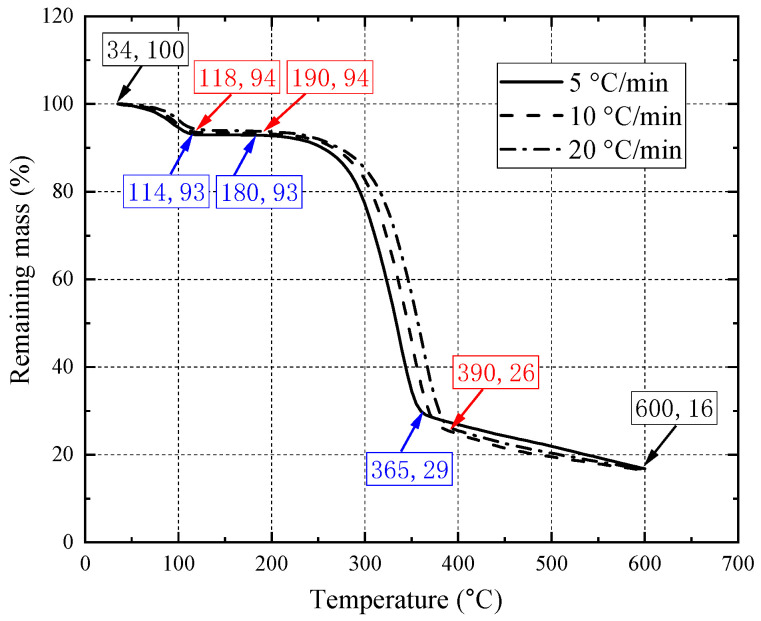
TGA test results at 5, 10 and 20 °C/min.

The TGA results of the Douglas-fir wood are shown in Figure 3. At the heating rate of 5 °C/min, the mass (highlighted in blue) of the sample significantly decreased from the initial temperature (approximately 34 °C) to 118 °C due to the moisture evaporation. Then the mass remained unchanged from 118 °C to 190 °C. Furthermore, due to the pyrolysis of the wood, the mass dramatically decreased from 94% at 190 °C to 29% at 365 °C. Subsequently, the mass gradually decreased to the end of the test. At 600 °C, the remaining mass of the wood sample is less than 20% of that at the room temperature. At the heating rate of 20 °C/min, the mass (highlighted in read) of the sample generally decreased slower than that at the heating rate of 5 °C/min. This resulted from the thermal lag in the sample. As a result, the TGA curve shifted toward right with the increasing heating rate.

### 3.2. Failure Modes

**Figure 4:**
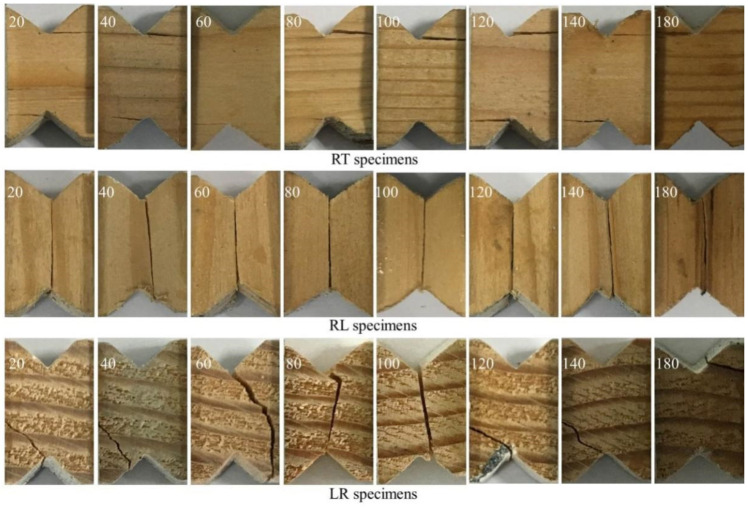
Shear failure modes of the specimens under elevated temperatures.

As shown in Figure 4, the failure modes of the specimens were generally dominated by the shear plane. RT specimens exhibited a typical non-linear load-displacement response until a first crack initiated at the root of the top notch and propagated along the grain. The ultimate failure was triggered by the second propagation of crack at the root of the bottom notch. Similarly, the ultimate failure of RL specimens was the cracking propagation between the notches along the grain. The failure of RL specimens was more sudden than that of RT specimens. The ultimate crack in LR specimen, however, initiated at the root of one notch and then developed with an angle (approximately 45°). Moreover, for some specimens (e.g., the LR specimens at 80 °C and 100 °C), the cracks were also formed along the radial direction. Similar shear failure modes were found in balsa wood in reference [24]. The temperature, however, seems to have a limited effect on the failure patterns. Nevertheless, the color turned dark with the increase in temperature from 140 °C to 180 °C. This can be attributed to the chemical reaction, i.e., the thermal degradation of cellulose, hemicellulose, and lignin.

### 3.3. Shear Stress-Strain Relationships

**Figure 5:**
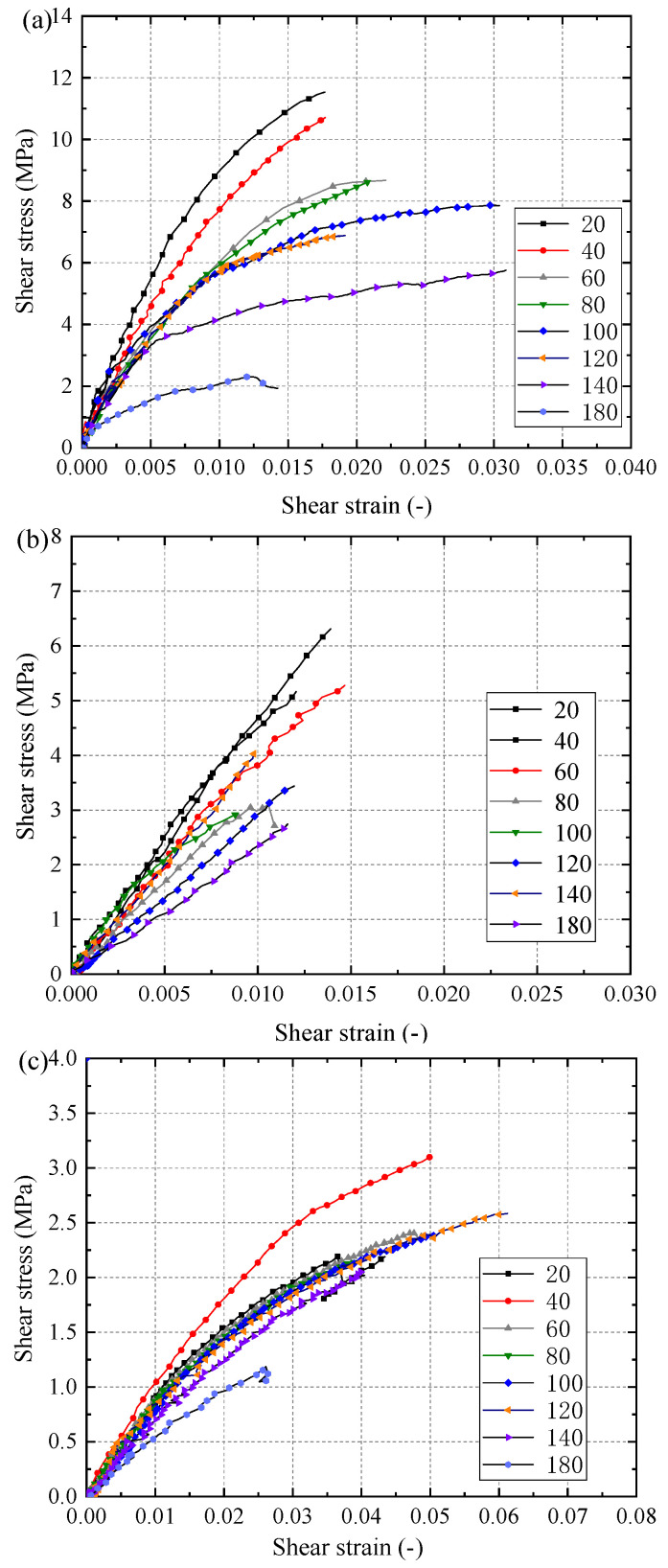
Typical shear stress-strain curves of specimens (**a**) RT; (**b**) RL; (**c**) LR.

Representative shear stress–strain curves in each group are plotted in Figure 5. The linear part of the stress-strain curve of the RT specimens is short at the initial stage. As the load increased, the curve exhibited obvious nonlinear characteristics. Moreover, the ultimate shear strain presented a tendency to increase first and then decrease with the increase of temperature. The ultimate shear strain increased from approximately 0.018 at 20 °C to 0.03 at 100 °C. After a clear reduction at 120 °C, it increased up to 0.031 at 140 °C and then reduced to 0.014 at 180 °C.

Comparatively, the stress–strain curves of RL specimens are linear during the whole loading process. The ultimate shear strain of RL specimens was distributed around 0.125, which was significantly smaller than that of RT group. This was because for RL specimens the shear force along the grain direction is mainly withstood by the matrix between the wood fibers, and the matrix has poor deformability due to the lack of wood fiber reinforcement. The shear stress–strain curves of LR specimens between 20 °C and 140 °C exhibited similar nonlinearity. Moreover, the shear strength in LR specimens was significantly lower than that of RT and RL specimens. However, the ultimate shear strain of LR is much higher than that of RT and RL. Consequently, the shear stiffness of LR specimens was much lower than that of RT and RL specimens. The ultimate shear strain at each target temperature was around 0.05, except for the shear strain of 180 °C group, which was 0.026 due to the severe thermal degradation.

### 3.4. Shear Strength versus Temperature

**Figure 6:**
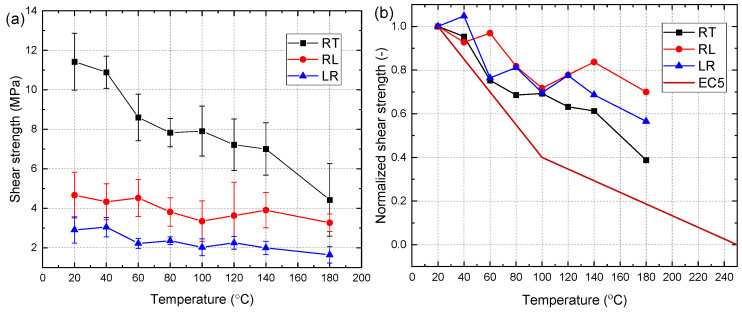
Shear strength of specimens: (**a**) Shear strength; (**b**) Normalized shear strength.

Table 1 and Figure 6a shows the average shear strength with standard deviation for each group of specimens at each target temperature. It can be observed that the shear plane had a great influence on the shear strength. At each target temperature, the strength of RT specimen was the highest, then RL specimen, and LR specimen was the lowest. At 20 °C, the average shear strength of RT, RL, and LR specimen was 11.4 MPa, 4.7 MPa, and 2.9 MPa, respectively. The difference in shear strength caused by three shear planes gradually decreased with the increase of temperature. At 180 °C, the average shear strength of RT, RL, and LR was 4.4, 3.3, and 1.6 MPa, respectively. It seems that the strength at each target temperature has significant standard deviation, which may stem from the spatial variability of the microstructure of the wood. Table 1 shows the coefficient of variation (C.V.) of the shear strength at each target temperature. The average value of the coefficient of variation of the RT specimens is approximately 0.17, which is very close to that of the LR specimens, whereas the RL specimens exhibited the largest average value of coefficient of variation (0.25).

Figure 6b shows the normalized shear strength (the shear strength retention rate with respect to the shear strength at 20 °C) of specimens with different shear planes. It can be seen that the strength of RT specimens exhibited a clear trend of decreasing with the increase of temperature. The maximum strength reduction of 0.6 was observed at 180 °C. For RL and LR specimens, the shear strength generally decreased as the temperature increased. However, a clear increment in shear strength was appeared in the range of 100–140 °C. The shear strength retention rate of the RL specimens increased from 0.72 at 100 °C to 0.84 at 140 °C while the LR specimens increased from 0.69 at 100 °C to 0.77 at 120 °C. This was because the elevated temperatures (100–140 °C) promoted the evaporation of bound water in the wood. Then, the dry lignin will be gradually hardened, leading to the increase of shear strength [18].

Figure 6b presents a comparison between the design curve for shear strength parallel to grain of wood at elevated temperatures suggested by EC 5 and the experimental curves of the shear strength retention rate in this study. It can be found that EC 5 is conservative for all the specimens with different shear planes. Moreover, it should be noted that the design curve in EC 5 is less conservative for the RT specimens than the RL and LR specimens.

### 3.5. Shear Modulus versus Temperature

**Figure 7:**
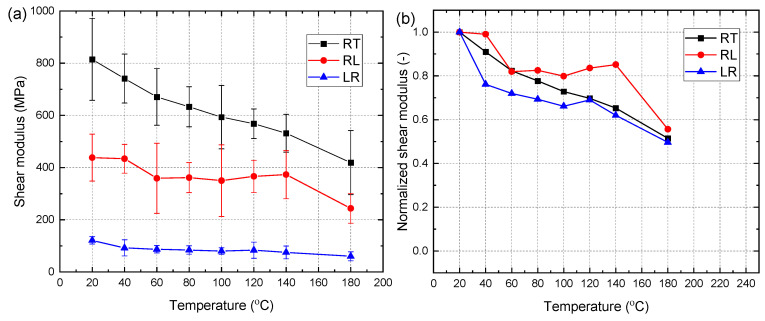
Shear modulus of specimens: (**a**) Shear modulus; (**b**) Normalized shear modulus.

Figure 7a depicts the shear modulus of all the specimens at different temperatures. Furthermore, the normalized shear modulus (shear modulus retention rate) of the specimens at each target temperature is shown in Figure 7b. It can be observed that the shear plane has a great influence on the shear modulus. The shear modulus of the RT specimens was the largest while that of the LR specimens was the lowest. Moreover, the shear modulus of all groups generally decreased with the increase of temperature. The retention rate of all groups decreased from 1.0 at 20 °C to approximately 0.55 at 180 °C. For RL and LR specimens, however, some fluctuation (less than 5.3%) of the shear modulus retention occurred between 100 °C and 140 °C. As describe before, this was due to the hardening of the dry lignin which significantly improved the shear properties. Moreover, the shear stiffness at each target temperature also exhibits significant standard deviation. As described before, this may stem from the spatial variability of the microstructure of the wood. Based on the coefficient of variation of the shear modulus in Table 1, it was found that the average value of the coefficient of variation of the RT specimens is approximately 0.17, which is smaller than those of LR and LR specimens (approximately 0.24).

## 4. Conclusions

This paper presents an experimental investigation on the shear behavior of Douglas-fir wood at elevated temperatures. Specimens with three types of shear planes were designed and tested from 20 to 180 °C. The effects of shear plane on the failure modes and shear cracks were presented. The temperature-dependent shear strength and shear modulus were obtained and discussed. Based on the experimental results, the following conclusions can be drawn:

1. The failure mode of the specimens is mainly affected by the shear plane. For RT specimens, cracks appeared at the root of the top notch and propagated along the grain. The RL specimens fractured along the grain between the two notches. The LR specimens mainly exhibited a crack initiated along the 45° direction from the notch. Moreover, the elevated temperatures showed limited influence on the failure mode.
2. The shear strength of the specimen at the same temperature significantly depends on the shear plane. The influence of shear plane on the shear strength gradually decreased with the increase of temperature. The reduction factor for shear strength given by Eurocode 5 is conservative for all the specimens with different shear planes. However, the design curve in Eurocode 5 is less conservative for the RT specimens than the RL and LR specimens.
3. The RT specimens exhibited the largest shear modulus while the LR specimens exhibited the lowest values at each target temperature. The shear modulus of all specimens generally decreased as the temperature increased. The shear modulus retention rate of all specimens decreased from 1.0 at 20 °C to approximately 0.55 at 180 °C.
4. Hardening of the dry lignin in the wood between 100 °C and 140 °C led to the improvement of shear strength and shear modulus, which was most obvious in RL specimens. The retention rate of the shear strength and shear modulus of the RL specimens increased 12.0% and 5.3%, respectively, when the temperature increased from 100 °C to 140 °C.

## Figures and Tables

**Table 1:** Shear strength, shear modulus and cracking details of the tested specimens.

Specimen	Shear Strength *τ* (MPa)	C.V.	Shear Modulus *G* (MPa)	C.V.	Cracking Orientation
RT-20	11.4	0.13	814.5	0.19	Parallel to grain
RT-40	10.9	0.07	741.2	0.13	Parallel to grain
RT-60	8.6	0.14	670.5	0.16	Parallel to grain
RT-80	7.8	0.09	632.8	0.12	Parallel to grain
RT-100	7.9	0.16	593.3	0.20	Parallel to grain
RT-120	7.2	0.18	567.8	0.10	Parallel to grain
RT-140	7.0	0.19	531.6	0.14	Parallel to grain
RT-180	4.4	0.41	419.1	0.29	Parallel to grain
RL-20	4.7	0.25	438.2	0.20	Parallel to grain
RL-40	4.3	0.21	434.0	0.13	Parallel to grain
RL-60	4.5	0.21	359.1	0.37	Parallel to grain
RL-80	3.8	0.19	361.7	0.16	Parallel to grain
RL-100	3.3	0.30	350.1	0.39	Parallel to grain
RL-120	3.6	0.46	366.3	0.17	Parallel to grain
RL-140	3.9	0.23	373.2	0.25	Parallel to grain
RL-180	3.3	0.13	243.9	0.23	Parallel to grain
LR-20	2.9	0.23	121.2	0.12	45° & radial direction
LR-40	3.0	0.16	92.3	0.34	45° & radial direction
LR-60	2.2	0.12	87.3	0.16	45° & radial direction
LR-80	2.3	0.08	84.1	0.19	45° & radial direction
LR-100	2.0	0.21	80.2	0.16	45° & radial direction
LR-120	2.3	0.15	83.7	0.37	45° & radial direction
LR-140	2.0	0.17	75.1	0.33	45° & radial direction
LR-180	1.6	0.25	60.2	0.29	45° & radial direction

## Data Availability

The data presented in this study are available on request from the corresponding author.
